# Growth pattern prediction of maxillary segments in infants with unilateral cleft lip and palate: a prospective in vivo study

**DOI:** 10.1007/s00784-025-06378-2

**Published:** 2025-05-26

**Authors:** Sarah Bühling, Cedric Thedens, Sara Eslami, Iulia Dahmer, Babak Sayahpour, Nicolas Plein, Lukas Benedikt Seifert, Robert Sader, Stefan Kopp

**Affiliations:** 1 Department of Orthodontics, Johann-Wolfgang Goethe University, Frankfurt, Germany; 2 Faculty of Medicine, Institute of Biostatistics and Mathematical Modelling, Johann-Wolfgang Goethe University, Frankfurt, Germany; 3 Clinic for Oral, Maxillofacial, and Facial Surgery, University Hospital Basel, Basel, Switzerland; 4 Clinic for Maxillofacial and Plastic Surgery, Johann-Wolfgang Goethe University, Frankfurt, Germany

**Keywords:** Cleft lip palate, Growth curve, Prospective study, Surface, Maxilla

## Abstract

**Purpose:**

The aim of the study was to identify the best prediction model for the growth pattern of the maxillary segments of infants with unilateral cleft lip and palate post birth and prior to the primary surgical cleft closure.

**Materials and methods:**

195 digital maxillary models of 50 infants with unilateral cleft lip and palate were collected during their preoperative alveolar molding therapy period. Intraoral scans were taken shortly after birth, at the monthly checkups and just before the surgical cleft closure at approximately 6 months of age. Surface measurements of maxillary segments were conducted using the diagnostic program OnyxCeph³™. For identifying the best fit for the growth pattern, mixed-effects regression models (fractional polynomials and B-splines) with the surface measurement as dependent variable, patient age (days) as predictor and the patient as random effect were fitted to the data. The best fit was selected according to the Akaike Information Criterium. A likelihood ratio test was performed for comparing the selected model with the intercept-only model.

**Results:**

The linear regression with mixed effects model showed the best fit for the total area, the area of the large segment and the area of the small segment. A highly significant association between the surface area and patient age was observed (*p* < 0.001). An increase of the area with time was estimated at 2.88 mm^2^ per day for the total area, 1.62 mm^2^ per day for the area of the large segment and 1.25 mm^2^ per day for the area of the small segment. The likelihood ratio tests indicated that the linear regression models were significantly better than the intercept-only models for all the measured areas (*p* < 0.01).

**Conclusion:**

The growth pattern for the maxillary segments of patients with unilateral cleft lip and palate in the preoperative period can be best predicted by using a linear regression model. The growth curve model developed by the present study can be used in future treatment planning of patients with unilateral cleft lip and palate.

**Supplementary Information:**

The online version contains supplementary material available at 10.1007/s00784-025-06378-2.

## Introduction

With the digitalization in dentistry and orthodontics, the digital contactless risk-free intraoral scan has been established and is increasingly replacing the conventional upper jaw impression [1]. Due to the reproducibility and accuracy, as well as the safety and simplicity the intraoral scanning has become an essential part of the pre-surgical orthopedic treatment of neonates with cleft lip and palate [2]. In addition, it enables recording the morphological changes in the maxillae of patients with unilateral cleft lip and palate risk-free and routinely at any desired intervals. If anatomically significant structures are not adequately represented, only the relevant section of the jaw needs to be rescanned, rather than repeating the entire digital impression. This allows for the generation of high-quality digital models.

The advances in the digital scanning technology have also resulted in computer-aided planning, digital manufacturing and computer-aided fabrication of maxillary plates in treatment of newborns with cleft lip and palate [3–5]. After digitally empirical blocking out the models, maxillary plates are digitally designed to cover the surface of the alveolar ridges up to the vestibulum, leaving out the soft-tissue radiating ligaments and the labial frenulum. The stepwise blocking out of the cleft area allows for passive and gradual narrowing of the cleft as the maxillary segments grow toward each other.

Craniofacial growth and changes in the size and shape of the maxilla are greatest between 0.4 and 1 year of age [6, 7]. During the growth process, shortly before and during early primary dentition, surface resorption begins in the maxilla, specifically in the premaxillary and neighboring nasal region, just inferior and lateral to the bony nasal opening [8]. The amount of maxillary growth that occurs during the various stages of early development remain largely unknown. Longitudinal, prospective studies of maxillary growth during infancy and early childhood are limited. A comprehensive understanding of early maxillary growth is essential for the effective treatment of newborns with cleft lip and palate, as well as the enhancement of the rehabilitation protocols. A standardized procedure for measuring models and close-meshed evaluation of early maxillary growth in this patient group has not yet been established.

Several studies have investigated morphological changes in the maxilla based on measurements taken from plaster casts [9–13], photographs [14] or specially made templates [15]. These techniques can lead to inaccurate results, not only in the positioning of the landmarks, but also in analyzing and transferring the data to the data to the computer [16]. Most of the previous analyses of casts to describe the morphological changes in the edentulous jaw of neonates with unilateral cleft lip and palate have relied on two-dimensional [9, 11] or three-dimensional registration of single landmarks on the mucosal relief [12, 13, 17]. However, given the complexity of craniofacial structures, and in particular of cleft lip and palates, more detailed measurements should be carried out. A number of scientific assessments have been able to demonstrate that surface measurements have been proposed in order to better quantify the effects of treatment [18, 19] Previous surface measurement evaluation studies have primarily relied on measurements of digitized plaster models taken at intervals of several months [16, 20–22].

This study introduces an innovative, non-invasive, precise, and reliable two-dimensional computer-aided method for longitudinal surface measurement of digital maxillary models in patients with unilateral cleft lip and palate using the OnyxCeph³™ analysis software (Image Instruments^®^ GmbH, Chemnitz, Germany). Thus, this study aimed to develop a growth curve model based on the surface growth of the maxillary segments of infants with complete unilateral cleft lip and palate prior to the primary surgical cleft closure.

## Materials and methods

### Study design and ethics

This monocentric prospective study aimed to develop a prediction model for the growth pattern of the maxillary segments of infants with complete unilateral cleft lip and palate prior to the primary surgical cleft closure. The study was approved by the ethics committee of the medical department of the J. W. Goethe University Frankfurt (Nr. 2023 − 1250). Informed consents were obtained from the parents and legal guardians of all patients.

### Patients

195 intraoral scans of fifty patients (40 male, 10 female) with a complete unilateral cleft lip and palate (33 left, 17 right) who meet the inclusion criteria were enrolled in this study.

### Inclusion criteria

The study included male and female patients who had undergone a full course of treatment atv J. W. Goethe University Frankfurt. These patients presented with a complete unilateral cleft of the lip, alveolus, and palate (UCLP), and had undergone presurgical infant orthopaedic treatment (PSIO) with maxillary plates in accordance with the Frankfurt concept. This concept includes a phase of PSIO therapy to orient the maxillary segments and reduce the cleft size prior to a single-step surgical closure of the cleft, performed at six months of age. In this concept, the newborns are fitted with the maxillary plate within 24–48 h after birth. Children are fitted with a new plate during the monthly check-ups, inorder to orient the maxillary segments and reduce the cleft size through gradual blockouts of the cleft. The surgical concept involves the closure of lip, alveolus, soft and hard palate through a Mucogingivo-periosteoplasty in a single step. Additionally, only patients with clearly identifiable surface on their maxillary intraoral scans were considered for inclusion in this study.

### Exclusion criteria

Patients with systemic diseases, syndromes or other deformities were excluded from the study.

### Sample size calculation

The sample size of 50 infants was selected based on the conservative “rule of 10 events per candidate predictor” – a widely used rule of thumb in regression analysis [23]. The rule recommends using a minimum of 10 measurements per estimated parameter when fitting a regression model at significance level of 5%. Since quadratic growth is typical for growth patterns in children, quadratic regression model with mixed effects was planned, considering the time as predictor for the surface of the maxillary segments and the patient as random effect. For this model 5 parameters are estimated: three for the quadratic function and two for the variances of the mixed model (intercept and slope).

### Data collection

Intraoral scans (IOS) were obtained from the patient’s maxillae postnatal (T0) and continued to be collected during the monthly check-ups (T1 = after 1 month; T2 = after 2 months; T3 = after 3 months; T4 = after 4 months; T5 = after 5 months; T6 = after 6 months; T7 = after 7 months) until just before cleft closure surgery with on average 6 months of age.

**Fig. 1:**
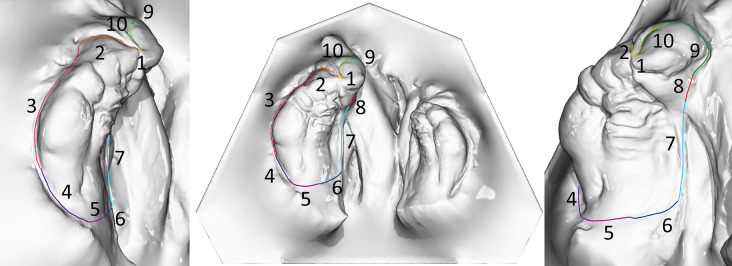
The boundary lines of the maxillary segments for the surface measurement of the large segment. 1 = yellow, 2 = orange, 3 = pink, 4 = purple, 5 = magenta, 6 = dark blue, 7 = light blue, 8 = red, 9 = dark green, 10 = light green

**Fig. 2:**
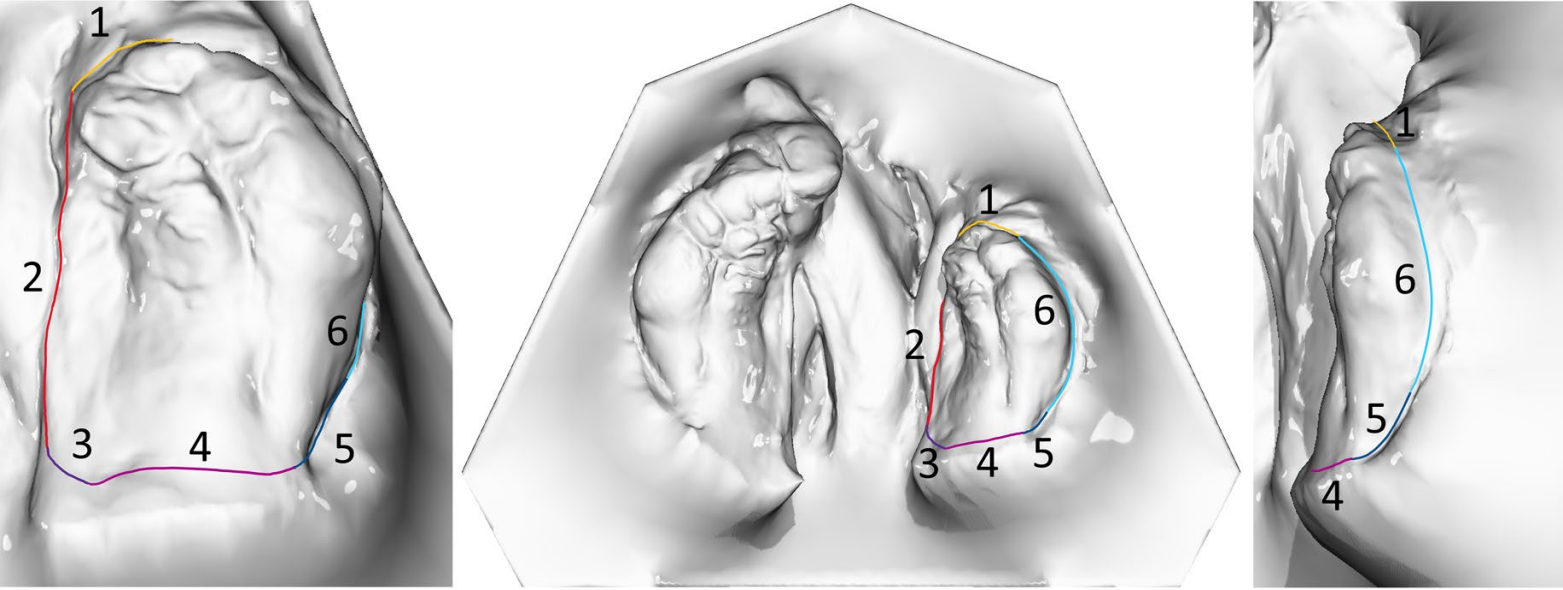
The boundary lines of the maxillary segments for the surface measurement of the small segment. 1 = yellow, 2 = red, 3 = purple, 4 = magenta, 5 = dark blue, 6 = light blue

**Fig. 3:**
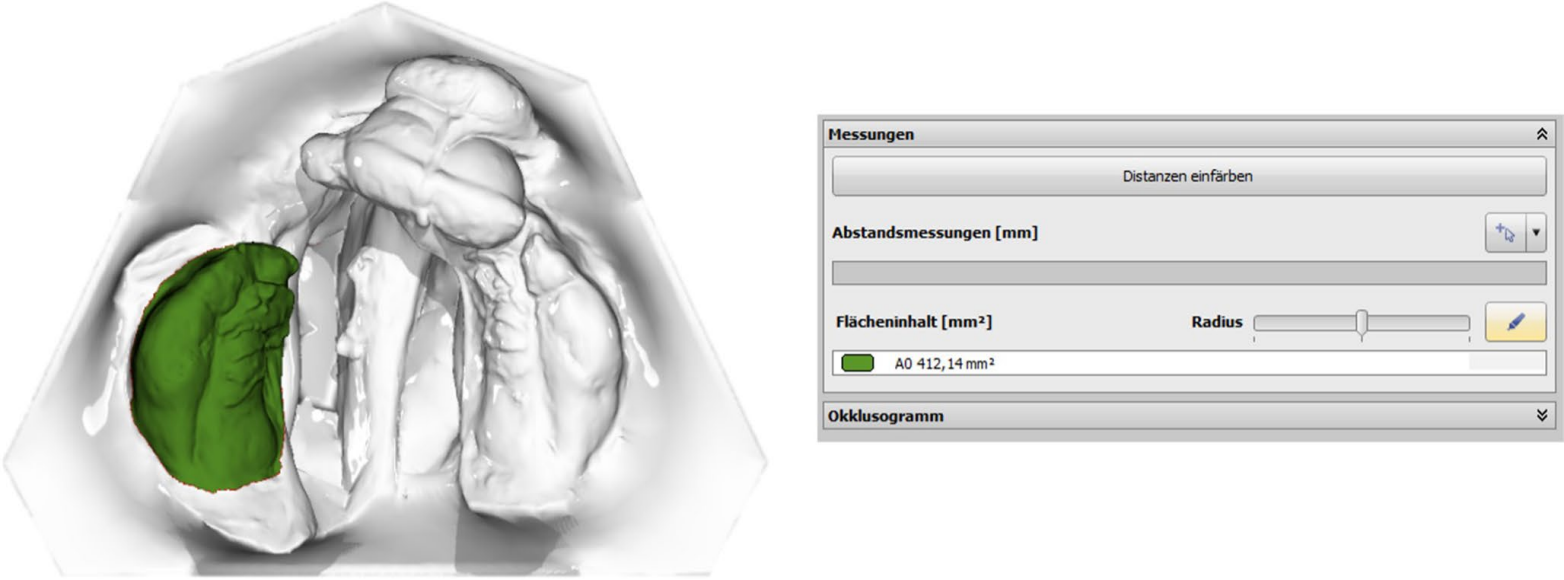
Surface measurement exemplary for the right segment in the OnyxCeph³™ software

The IOS were obtained using the TRIOS 4 wireless intraoral scanner (3shape, Copenhagen, Denmark) following a standardized protocol [24]. Afterwards, the Stl. Datei of patients’ maxilla were imported in OnyxCeph³™ software (Image Instruments, Chemnitz, Germany). The surface of the digital cleft palate models were precisely evaluated using the module ‘Inspect 3D’ of OnyxCeph³™ software. This module enables the users to define the surface of a maxillary segment by setting the measurement points according to the ‘jaw borders’ presented in Figs. 1–2 and Table 1 [25]. The surface area of the defined maxillary segment (Fig. 3) were then determined in the module ‘Inspect 3D’. The surface measurement boundary lines and their definitions are shown in the Tables 1 and 2 and Figs. 1 and 2. These boundaries have been determined in cooperation with oral and maxillofacial surgery department and anatomy department of J. W. Goethe University Frankfurt. Subsequently, the surface measurements of the maxillary segments were performed by one examiner.The respective surfaces were measured for both maxillary segments using the Inspect 3D tool of the OnyxCeph³™ software (Fig. 3).Using the modules “Model Alignment” and “Evaluation” of the software, each measurement was saved as X, Y and Z coordinates. Vector calculations determined distances and angles between points in three spatial dimensions. Surface areas of selected regions were calculated by summing the selected polygon areas. Custom patches were developed within the software to support this measurement method, allowing for streamlined anatomical landmark analysis through the “Measurement” module.

**Table 1:** The boundary lines of the maxillary segments for the surface measurement for the large segment

	Landmarks (boundary line)	Description
1.	Region of the labial frenulum/incisal point	The borderline is positioned at the incisal point, excluding the labial frenulum.
2.	Transition from the lip to the alveolar ridge (to the right of the labial frenulum)	The deepest indentation in the right half of the premaxilla, distinguishing it from the adjacent lip.
3.	Mucobuccal fold in the vestibule	The deepest indentation of the alveolar ridge in the vestibular area.
4.	Sulcus palatinus lateralis	The transition from the vestibule to the dorsal region of the tuber point.
5.	Tuber boundary	The boundary line is located dorsal to the tuber point.
6.	Sulcus palatinus medialis	The transition from the dorsal tuber region toward the vomer.
7.	Margo medialis processus palatinus ossis maxillaris	The mucosal fold that manifests as a bony prominence of the jawbone.
8.	Transition to the premaxilla	The shortest connection between the previous boundary of the bony jawbone prominence and the left area of the premaxilla.
9.	Lateral boundary of the premaxilla	Visible boundary of the bony portion of the left side of the premaxilla.
10.	Transition from the lip to the alveolar ridge (to the left of the labial frenulum)	The deepest indentation in the left half of the premaxilla, distinguishing it from the adjacent lip.

**Table 2:** The boundary lines of the maxillary segments for the surface measurement for the small segment

	Landmarks (boundary line)	Description
1.	Anterior cleft tip	Area of the jaw prominence in the anterior region, beneath the anterior cleft tip.
2.	Margo medialis processus palatinus ossis maxillaris	The mucosal fold that appears as a bony prominence of the jawbone.
3.	Medial palatine sulcus	The transition from the medial margin of the palatine process of the maxilla toward the dorsal tuber region.
4.	Tuber boundary	The boundary line is located dorsal to the tuber point.
5.	Sulcus palatinus lateralis	The transition from the dorsal region of the tuber point toward the vestibule.
6.	Mucobuccal fold in the vestibule	The deepest indentation of the alveolar ridge in the vestibular area.

### Statistical analysis

The statistical evaluation was carried out with under the supervision of the Institute for Biostatistics and Mathematical Modeling of the Faculty of Medicine at J. W. Goethe University Frankfurt.

Statistical analyses and graphical representations were performed with R version 4.4.1, packages “nlme” (version 3.1–167), “mfp” (version 1.5.4.1) and “splines” (version 3.6.2), (R Foundation for Statistical Computing, Vienna, Austria) and RStudio version 2024.09.1 (Posit Software, PBC).

The data was first presented descriptively. Then mixed-effects regression models with time (days) as predictor and the patient as a random effect (intercept and slope) were fitted to the data for the total area, the area of the large and the area of the small segment separately. The fitted models included fractional polynomials and B-spline models. Linear regression as well as the quadratic and the squared root regression were tested as potential fits within the fractional polynomials model. The B-spline model was considered in order to include possibly more complex nonlinear relationships between the surface and time.

The best fit was selected according to the Akaike Information Criterium (AIC) [26]. The AIC score is a measure of the relative quality of a statistical model fit for the observed data. It estimates the relative amount of information that is lost when describing the data with a given model. Therefore, when comparing different candidate models, a lower AIC score indicates a better fit. This score is only useful when comparing models and does not measure the absolute quality of a specific model.

The AIC score is based on the log-likelihood of the model (a measure of the discrepancy between observed values and the values expected under the model in question) and includes a penalty term that characterizes the complexity of the model (twice the number of parameters estimated by the model).

Furthermore, likelihood ratio tests were performed for comparing the selected model with the intercept-only model for each of the three areas.

To confirm measurement reliability, intra-rater reliability was assessed by remeasuring 20 randomly selected intraoral scans two weeks apart. The intraclass correlation coefficient (ICC) demonstrated excellent to good reliability for total surface area, as well as for the large and small segment measurements (ICC > 0.80).

For all analyses the significance level was set at 5%.

## Results

195 surface measurements were included. The number of measurements per patient varied between 1 and 7 with a median of 4 measurements per patient.

Table 3 shows the surface growth per day (mean + SD) computed as difference between the first and last measurement of the patient divided by the age (days) at the last measurement. The values for the total area and for the two segments are presented as absolute values and in percentage. The growth of small and large segments as well as total maxillary surface are presented in Videos 1–3.

**Table 3:** Surface growth per day (mm^2^) of the total area, the large and the small segment

	Total area (mean ± SD)	Area of the large segment (mean ± SD)	Area of the small segment (mean ± SD)
Growth per day (mm^2^)	2.90 ± 0.91	1.64 ± 0.56	1.26 ± 0.47
Growth per day (%)	0.28 ± 0.09	0.26 ± 0.01	0.31 ± 0.12

SD = standard deviation

**Fig. 4:**
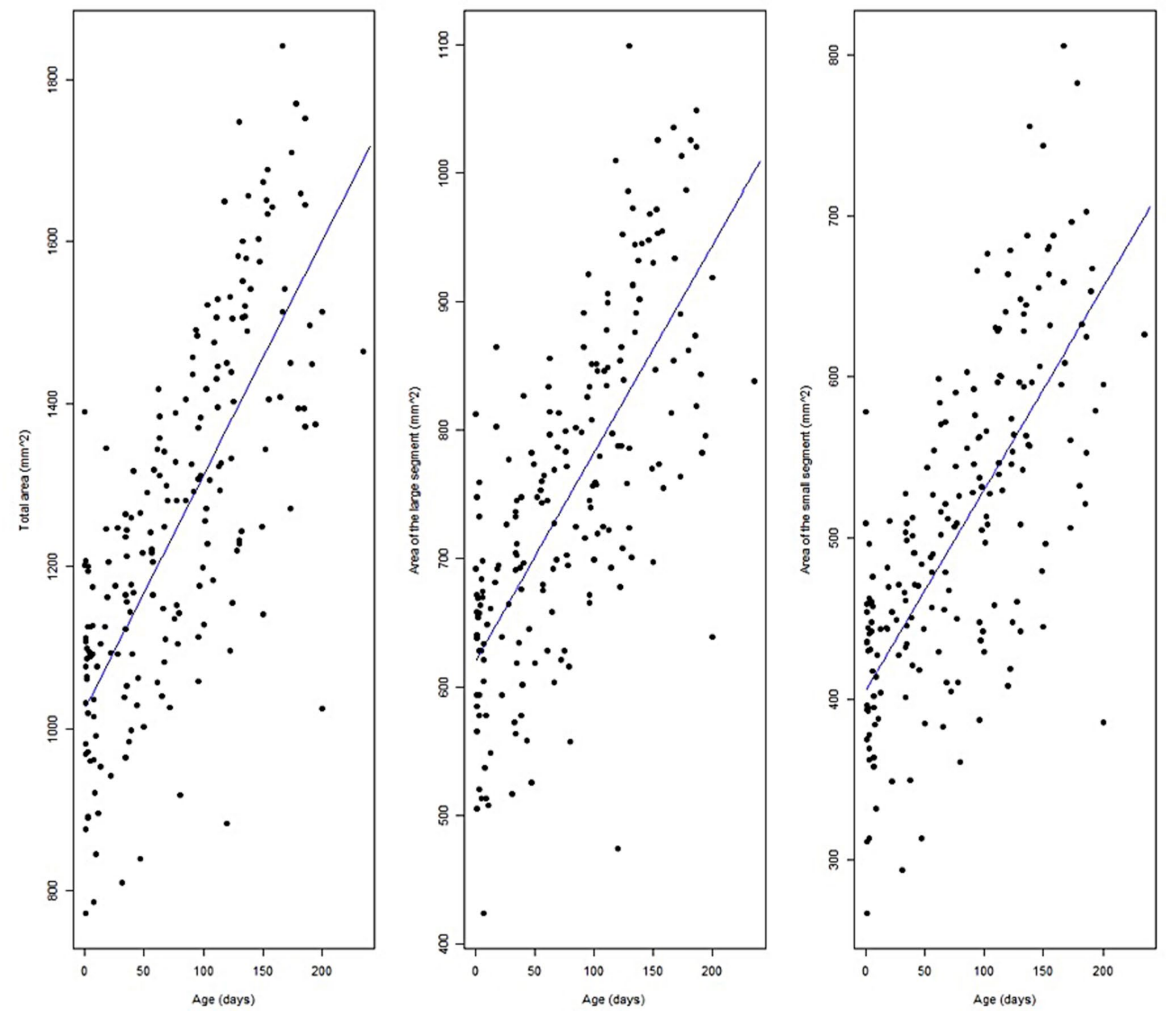
Fitted linear prediction models of growth pattern for the three areas (total area, large segment and small segment)

The fractional polynomials model selected the linear model as optimal fit for all the three areas (total, large segment and small segment) (Fig. 4). In each case, this model had lower AIC values than the B-spline models (Table 4). Even though the differences in the AICs are low, we retained the linear model as best fitting model since a simpler model is preferable to a more complex one including more parameters.

**Table 4:** AIC-values for the fitted models

Model	Total area	Area of the large segment	Area of the small segment
Fractional-polynomials-regression (linear model)	2222.35	2074.11	1952.59
B-splines-regression	2223.82	2077.72	1956.21

**Fig. 5:**
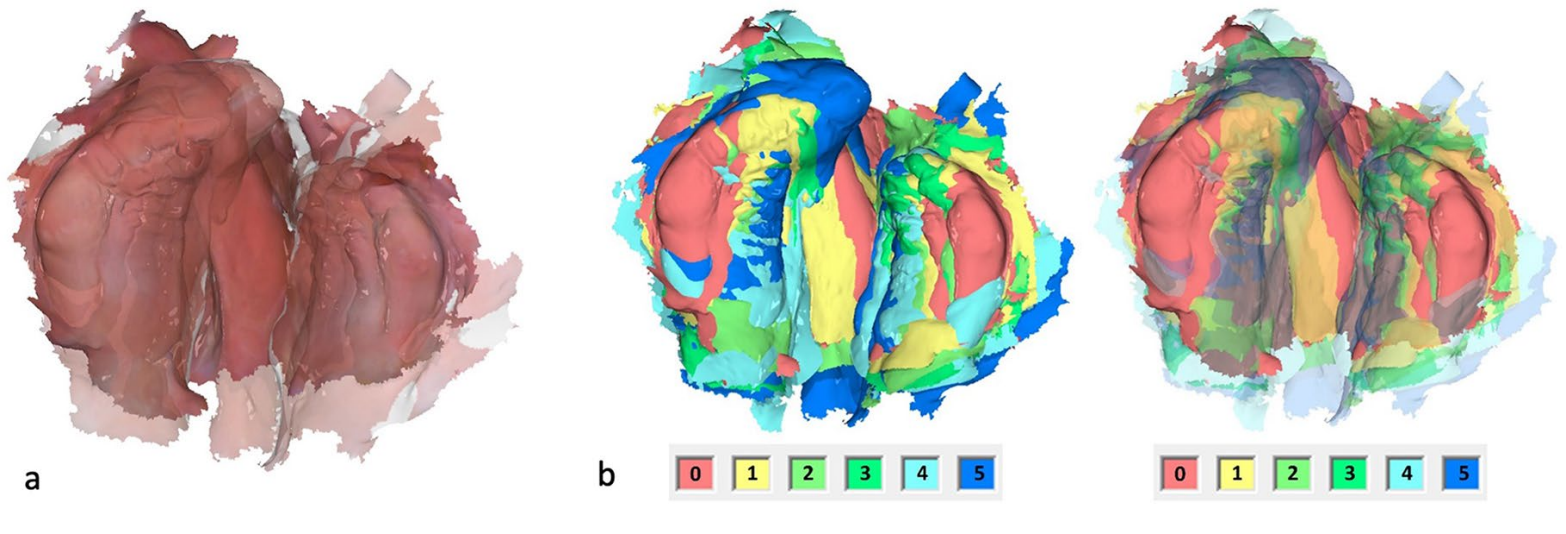
Surface enlargement of the complete maxilla over the entire preoperative treatment period. **a** Postnatal and preoperative IOS overlay. **b** Overlay of six IOS, entire jaw. Left without transparency, right with increasing transparency, in ascending order

**Fig. 6:**
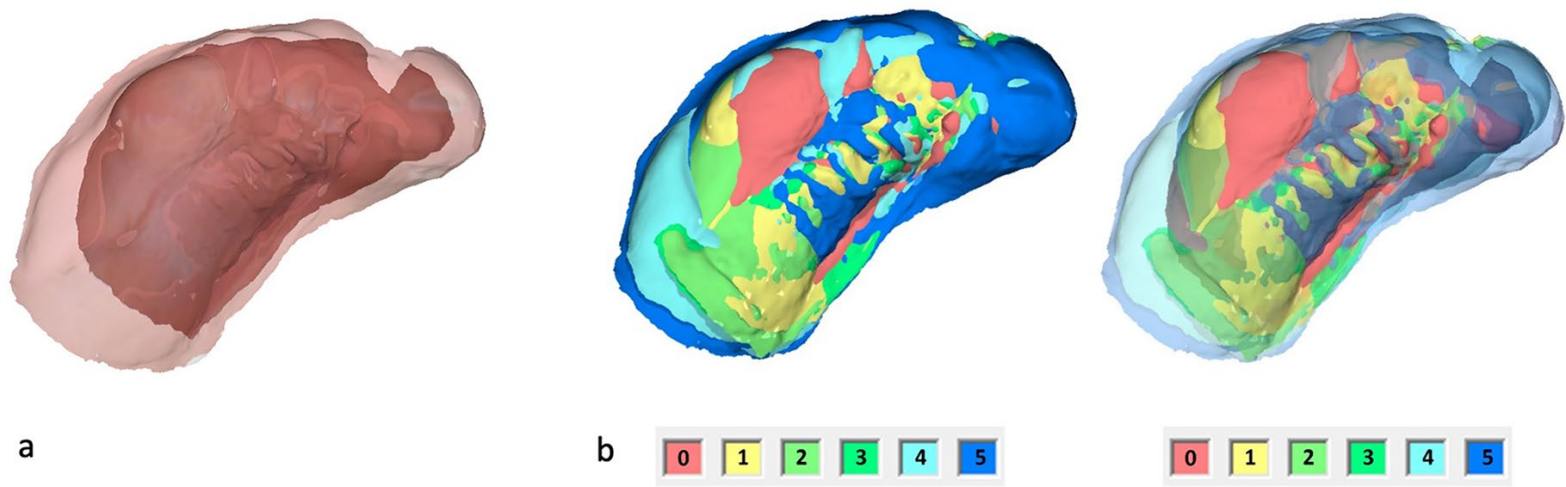
Surface enlargement of the large maxillary segment over the entire preoperative treatment period. **a** Postnatal and preoperative IOS overlay. **b** Overlay of six IOS, entire jaw. Left without transparency, right with increasing transparency, in ascending order

**Fig. 7:**
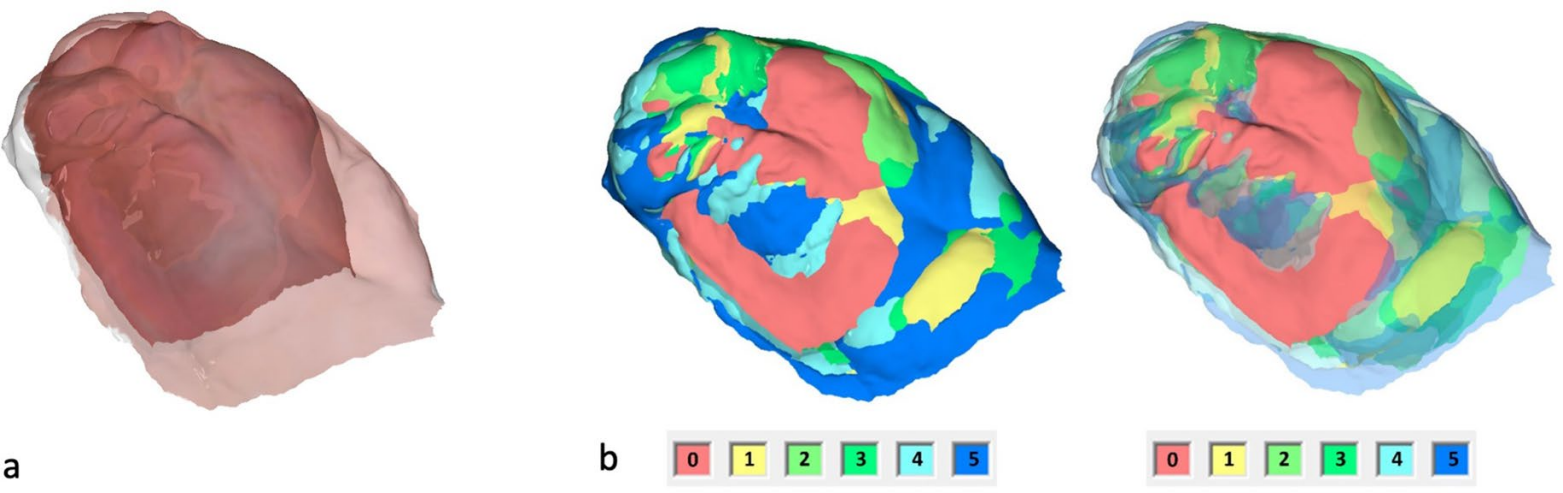
Surface enlargement of the small maxillary segment over the entire preoperative treatment period. **a** Postnatal and preoperative IOS overlay. **b** Overlay of six IOS, entire jaw. Left without transparency, right with increasing transparency, in ascending order

Overall, the model fit analysis identified the linear model with mixed effects (patient as random factor) as the best fit for the surface growth pattern for all three areas (total, large segment and small segment). The surface areas are estimated to grow with increasing patient age, time being a highly significant predictor for the surface growth in all three cases (*p* < 0.001). The detailed results of the linear regressions are presented in Table 5 and are visualized in Figs. 5, 6 and 7.

**Table 5:** Summary of the results of the linear regressions

	Regression coefficient	*p*-value
**Total area**		
Intercept	1024.07	< 0.001
Age (days)	2.88 (95%-CI: (2.65, 3.11))	< 0.001
**Area of the large segment**		
Intercept	619.86	< 0.001
Age (days)	1.62 (95%-CI: (1.49, 1.75))	< 0.001
**Area of the small segment**		
Intercept	404.87	< 0.001
Age (days)	1.25 (95%-CI: (1.13, 1.38))	< 0.001

For all three areas the likelihood-ratio-tests showed highly significant differences between the null-model and the linear model (*p* < 0.001).

## Discussion

The primary objective of this study was to fit a growth curve model for the surface growth of the maxillary segments of infants with complete unilateral cleft lip and palate. The linear model was chosen as best fit to describe the early maxillary growth from birth to the time of the surgical cleft closure. The study showed that significant surface arch growth in both maxillary segments of patients with unilateral cleft lip and palate could be stimulated within the presurgical orthodontic intervention period, with time having a significant influence.

### Comparison with previous studies

Many authors have already addressed the measurement of maxillary segments in cleft lip and palate patients. However, whether the analyses were two [9, 11] or three dimensional [12, 13, 17, 27], they were largely based on the measurement of individual selected landmarks, measuring distances and angles between them. Complex anatomical structures, such as those in patients with cleft lip and palate, are best characterized through more detailed and comprehensive analyses, such as surface evaluation. Studies, including those by Darvann et al. [19], have demonstrated that computer-aided surface measurements can minimize errors and provide highly accurate assessments. Jorge et al. conducted a narrative review highlighting the latest non-invasive diagnostic techniques for evaluating maxillary arches in patients with orofacial clefts, including surface measurement [28]. Previous surface measurement evaluation studies have primarily relied on measurements of digitized plaster models taken only at limited timepoints [16, 20–22] Ambrosio et al. [20] investigated digitized dental models of children with UCLP before cheiloplasty, after cheiloplasty and after palatoplasty. The evaluated surface area was delimited from the alveolar ridge to the intertuberosity distance, neglecting the vestibular tooth germ-bearing alveolar parts vestibular of the palatal gingival grooves. Consistent with our results, it was shown that there is a significant increase in the maxillary segment surface area over time. Bruggink et al. [21] measured the surface of digitized plaster casts from 28 patients with unilateral cleft lip and palate at two timepoints, defining the palatal area as the area between several defined anatomical landmarks, not representing the actual surface of the maxillary segments. In this study, no significant increase in maxillary segment surface area was observed between the two control periods. Hoffmannova et al. [22] analyzed digitized dental casts from 56 patients with unilateral cleft lip and palate, taken shortly after birth and at 10 months of age. They defined the transverse boundary lines for measuring the maxillary segment surfaces using the mesial and distal margins of the canine swelling, as well as the distal margin of the molar swelling. The surface areas of both maxillary segments were overlayed digitally and compared at two time points. The analysis revealed significant growth in the anterior portions of both segments, leading to a reduction in the width of the anterior cleft. Growth was also observed in the posterior sections of both segments, with more pronounced development on the non-cleft side. A detailed quantitative analysis was not carried out. De Menezes and colleagues [16] conducted a study on 96 digitized palatal casts from 32 neonates with UCLP, taken at three different time points. Their surface measurement landmarks were positioned at the junction between the alveolar process and the maxillary bone, enabling the assessment of both the palatal and vestibular regions of the maxillary segments. According to our findings they demonstrated a significant influence of both alveolar surface area and time. According to current scientific literature [18, 29] in our scientific research the large and small segments grow a comparable amount. However, our study demonstrated that the small segment shows a slightly greater percentage increase in surface area.

### Clinical implications

The advances in the digital scanning technology have resulted in computer-aided planning, digital manufacturing and computer-aided fabrication of maxillary drinking plates [3, 4]. These digital methods not only improve patient comfort but also eliminate risks associated with conventional impression materials, such as aspiration or tissue irritation. Moreover, intraoral scanning provides highly accurate digital impressions, reducing the risk of material-related errors such as distortion, bubbles, or tearing that can occur with traditional alginate impressions and thus increase the accuracy and precision of the dental models to be measured [30, 31]. Surface growth measurements provide a detailed evaluation of how the maxillary segments expand and reshape, particularly in regions affected by the unilateral cleft lip and palate. These measurements can help identify asymmetries, monitor post-surgical healing, and assess the impact of PSIO. For instance, assessing whether surface growth occurs uniformly across the alveolar ridges of the maxilla is essential for evaluating the effectiveness of treatments aimed at achieving symmetrical and harmonious jaw development. Uneven growth may indicate the need for adjustments in treatment protocols, such as modifications to preoperative orthodontic appliances or surgical planning. The maxillary plates used in the PSIO are digitally crafted to accurately replicate the contours of the maxillary segments.

### Forward planning in PSIO therapy

Knowledge of the monthly growth-related changes in the surface of the maxillae of patients with complete unilateral cleft lip and palate is essential for the forward-looking digital planning of drinking plates to ensure the most appropriate plate fit and therapeutic success. A precise understanding of in which areas and how much the surface changes is essential. As digital measurement methods continue to evolve, it would be beneficial to enable the measurement of surface changes in specific jaw sections. This would allow for a more precise quantification of whether jaw surface growth is evenly distributed between the anterior and posterior regions. The anterior and posterior alveolar ridges contain the deciduous and the permanent teeth. The different stages of dental development may influence the increase in these area variables. It is also important to be aware that the growth predictions presented in this study are based on measurements of soft tissue, not bony structures. Moreover, the direction and degree of maxillary jaw growth are influenced by the severity of the malformation and the particular type of cleft. It is essential to highlight that these factors can vary significantly among individuals. Therefore, it is essential to carry out additional digital anthropometric studies of intraoral scans, particularly focusing on the postoperative period and patients with bilateral cleft lip and palate.

The results obtained from this study are highly relevant in both clinical settings and scientific research on unilateral cleft lip and palate (UCLP). We wanted to assess a feasible and useful tool for the digital assessment of more complex measurement of the maxillary arches in patients with cleft lip and palate, enabling longitudinal assessments of jaw surface changes and therefore facilitating comparisons across different time points to track growth trends, especially in long term treatment evaluation. Moreover, we fitted a growth curve model for the surface growth of the maxillary segments of infants with complete unilateral cleft lip and palate that can be used in the forward digital treatment planning of newborns with unilateral cleft lip and palate.

## Limitations

The demographic of the intraoral scans as well as the participants were quite narrow in this study, what affects the generalizability of the results adversely. While cleft lip and palate is typically more common in males, with a reported male-to-female ratio of approximately 2:1, our study sample shows a greater gender imbalance. This should be taken into account as a limitation when interpreting the results. Data were analyzed per protocol, which always carries a risk for bias. According to a studie by Braumann et al. [32] the success of a model analysis depends not only on the quality of the model but also on the individual experience of the examiner. Therefore, intraoral scanning, post-processing of the digital model surface, setting of anatomical measurement and reference points were performed by the same person for all models to ensure uniformity. Despite the benefits of surface growth analysis, several challenges persist. High-quality digital impressions require advanced intraoral scanners and compatible software, which may be cost-prohibitive for smaller practices. Additionally, the depth of field and resolution of scanners must be optimized to accurately capture the complex anatomy of cleft-affected regions, particularly in deep or narrow areas between maxillary segments. Another limitation is the lack of standardized protocols for surface measurement in cleft lip and palate patients. While some studies have explored surface area changes, there is a need for consistent reference points, measurement techniques, and analysis criteria to ensure comparability across clinical settings. Variations in scanner types, patient cooperation, and software capabilities can also influence the accuracy and reliability of surface measurements.

## Conclusions

This study successfully developed a linear growth curve model to describe the surface expansion of maxillary segments in infants with complete unilateral cleft lip and palate during the preoperative period. The model demonstrated that significant surface growth occurs in both segments, particularly under the influence of time and presurgical orthopedic intervention.

Compared to earlier studies that primarily relied on landmark-based measurements, our approach offers a more comprehensive and surface-oriented method, improving the accuracy of growth assessment. These findings support the use of digital surface analysis as a valuable tool in early treatment planning and monitoring, particularly for forward-looking PSIO strategies. As digital intraoral scanning and modeling technologies advance, their integration into cleft care holds promise for more individualized, precise, and dynamic therapy protocols.

Leveraging artificial intelligence and machine learning to enhance surface measurement accuracy, identify growth patterns, and predict outcomes based on longitudinal data is also conceivable.

## Electronic supplementary material

Below is the link to the electronic supplementary material.

Supplementary Material 1: Video 1. Visualization of the large segment growth.

Supplementary Material 2: Video 2. Visualization of the small segment growth.

Supplementary Material 3: Video 3. Visualization of the total maxillary growth.

## Data Availability

The data is available upon request from the corresponding author.
